# Progress in coverage of bed net ownership and use in Burkina Faso 2003–2014: evidence from population-based surveys

**DOI:** 10.1186/s12936-017-1946-1

**Published:** 2017-07-28

**Authors:** Sekou Samadoulougou, Morgan Pearcy, Yazoumé Yé, Fati Kirakoya-Samadoulougou

**Affiliations:** 1 Pôle Epidémiologie et Biostatistique, Institut de Recherche Expérimentale et Clinique (IREC), Faculté de Santé Publique (FSP), Université catholique de Louvain (UCL), Clos Chapelle-aux-champs 30, bte B1.30.13, 1200 Bruxelles, Belgium; 2 Spatial Epidemiology Lab (SpELL), Université Libre de Bruxelles, Brussels, Belgium; 3 ICF, 530 Gaither Road, Suite 500, Rockville, MD USA; 4 Centre de Recherche en Epidémiologie, Biostatistiques, et Recherche Clinique, Université Libre de Bruxelles, Brussels, Belgium; 5 Plate-Forme Biostatistiques, Pôle Santé, Université Libre de Bruxelles, Brussels, Belgium

**Keywords:** Bed net, LLIN ownership gap, LLIN use gap, LLIN access gap, Behavioural failure, Malaria

## Abstract

**Background:**

Use of insecticide-treated bed nets (ITNs) is the cornerstone of malaria prevention. In 2010 and 2013, the Burkina Faso Government launched mass distribution campaigns of ITNs to increase coverage of ownership and use in the country. This study assessed the progress towards universal bed net coverage in Burkina Faso.

**Methods:**

The authors used data from the Burkina Faso 2003 and 2010 Demographic and Health Surveys (DHS), the 2006 Multiple Indicator Cluster Surveys (MICS) and the 2014 Malaria Indicator Survey (MIS). For each survey, the authors computed key malaria prevention indicators in line with recommendations from the Survey and Indicator Task Force of the Roll Back Malaria Monitoring and Evaluation Reference Group. The trends over a decade was assessed by calculating percentage point change between 2003 and 2014.

**Results:**

At national level, the proportion of households owning at least one ITN increased substantially from 5.6, 95% CI (4.7, 6.5%) in 2003 to 89.9% (88.5, 91.2%) in 2014, with low heterogeneity between regions. The proportion of households owning at least one ITN per two people increased significantly from 1.8% (1.4, 2.3%) in 2003 to 49.2% (47.3, 51.0%) in 2014. ITN use in the general population increased from 2.0% (1.6, 2.3%) in 2003, to 67.0% (65.3, 68.7%) in 2014. A similar trend was observed among children under the age of  five years, increasing from 1.9% (1.5, 2.4%) in 2003 to 75.2% (73.2, 77.3%) in 2014, and among pregnant women, increasing from 3.0% (1.9, 4.2%) in 2003 to 77.1% (72.9, 81.3%) in 2014. The intra-household ownership gap was 67.0% (61.5, 72.4%) in 2003, but decreased significantly to 45.3% (43.6, 47.1%) in 2014. The behavioural gap, which was relatively low in 2013 with only 20.0% of people who had access to an ITN but were not using it, further decreased to 5.9% in 2014.

**Conclusion:**

Burkina Faso made considerable progress in coverage of ITN ownership, access and use between 2003 and 2014, as a result of the two free mass distribution campaigns in 2010 and 2013. However, ITN coverage remains below the national targets of 100% for ownership and 80% for use. The results of 90% of ownership and 67% of use confirm that free mass distribution campaigns of ITNs are effective; however, there is room for improvement to reach and maintain optimal coverage of ITN ownership and use.

## Background

Insecticide-treated bed nets (ITNs) are effective tools for malaria control [1]. Meta-analyses have shown that ITNs are associated with an 18% reduction in child mortality [2], 51% decrease in uncomplicated malaria incidence and 17% reduction in parasite prevalence in children [3]. In the past decade, the rapid scale-up of bed nets in sub-Saharan Africa (SSA) contributed to the significant decline of malaria burden in the region [4, 5]. Sustaining high coverage of this intervention is critical to decrease further the burden of the disease and reach the long term-goal of malaria elimination. It is estimated that a minimum of 150 million ITNs per year are needed to maintain a constant pool of 450 million functioning ITNs to protect individuals at risk in SSA [6].

Increasing ITN coverage has been achieved using various distribution strategies, including social marketing [7–9], free distribution to target vulnerable groups (pregnant women and children under the age of five) through antenatal care (ANC) or immunization campaigns [7, 8, 10–12], and more recently, free, universal, population-based distribution campaigns targeting the general population [7, 8, 10, 12–18]. The World Health Organization (WHO) recommends to distribute free or subsidize bed nets as the best way to ensure full coverage [19].

In 2001, a nationwide survey in Burkina Faso estimated that only 12.4% of children under the age of five were sleeping under a net, compared to 23.2% in 2005. Among pregnant women this proportion was 10.0% in 2001 and 27.5% in 2005 [20]. To rapidly increase coverage of ITN ownership and use, particularly among vulnerable groups, the Government of Burkina Faso initiated a first national-scale, free distribution campaign of ITNs in 2010. The aim of the campaign was to ensure that households had access to at least one ITN for every two people through the distribution of about eight million long-lasting insecticidal nets (LLINs). Moreover, in 2013, the national malaria control programme (NMCP) launched the second free LLIN distribution campaign to scale-up the coverage of ITNs in the country. This campaign aimed to ensure that 100% of households owned at least one ITN, and reach 80% ITN use by 2015. In 2014, the Burkina Faso Government decided to conduct the first Malaria Indicator Surveys (MISs) to assess coverage and impact of scaled-up malaria interventions. MISs were developed by the Roll Back Malaria (RBM) Monitoring and Evaluation Reference Group (MERG) with the aim to help national ministries of health collect key and timely information on malaria control at national level [21]. As Burkina Faso aims to achieve universal coverage with LLINs, this paper assessed the progress and gaps in coverage of bed net ownership and use based on RBM/MERG-recommended indicators [21].

## Methods

The authors analysed regional trends of ITNs ownership, access and use indicators in Burkina Faso over 11 years. These indicators were computed using data from the 2003 and 2010 Demographic and Health Surveys (DHS) [22, 23], 2006 Multiple Indicators Cluster Survey (MICS) [24] and the first national MIS 2014 [25]. At the time of these surveys, Burkina Faso was divided into 13 administrative regions.

### Data from Demographic and Health Survey 2003 and 2010

DHS 2003 (between June and December 2003) and DHS 2010 (between May 2010 and January 2011) were conducted during the high transmission season. DHS was designed to obtain national and regional estimates for malaria indicators. The DHS surveys followed a two-stage selection process in which a random sample of clusters was first selected from the most recent national sample frame. In the second stage, all households were listed and the final list of households selected by systematic random sample. In the Burkina Faso DHS, the sample was selected in two stages, stratified by place of residence (urban and rural) with enumeration areas (EAs) as the first-stage sampling units, and households as the second-stage sampling units. Further details are provided in the DHS reports [22, 23].

### Data from MIS 2014

The MIS data were collected from October to November 2014 (at the end of the high transmission season), using the standard malaria indicator questionnaires developed by the RBM and the DHS Program. The dataset consists of malariometric information, demographic characteristics and socio-economic status on a nationally representative sample of 6448 households from 252 clusters, of which 52 are in the urban areas. These clusters were derived from a stratified two-stage cluster design. A detailed description of the sampling strategies is documented in the final report of the 2014 Burkina Faso MIS [25].

### Data from MICS 2006

Multiple Indicator Cluster Surveys are typically carried out by government organizations with the support and assistance of UNICEF to fill data gaps for monitoring the children and women wellbeing. The Burkina Faso MICS conducted from March to June 2006 used a two-stage stratified sample design. At the first stage of sampling, 198 census EAs (197 visited) were selected. The clusters in each region were selected using systematic sampling with probability proportional to their size. A complete household-listing exercise covering all EAs in the 2003 Burkina Faso DHS was carried out. At the second stage, a systematic sampling of households was selected based on this list. For the 2006 Burkina Faso MICS, 30 households per EA were selected per rural EA, 32 (in Ouagadougou, the capital city) to 36 households per urban EA. Due to the fixed sample size per EA, the disproportional number of EAs and different sample sizes selected per EA among regions, the household sample is not self-weighting at the national level. A more detailed description of the sample design can be found elsewhere [24].

### Indicators

Ownership, access, use, and gap indicators were calculated from the datasets of households and individual household members, as recommended by MERG [21] (Table 1).

**Table 1:** RBM/MERG-approved indicators used

Indicator	Numerator	Denominator
Ownership		
Proportion of households in the survey with at least one ITN (P1)	Number of households owning at least one ITN	Number of households in the survey
Proportion of households with sufficient access to ITN (P2)	Number of households owning at least one ITN for every two household members	Number of households in the survey
Proportion of population with access to ITN within the household (P3)	Potential number of household members protected by the ITN (i.e., number of ITN owned multiplied by two), or number of de facto household members in the household, whichever was the lowest	Population in the survey
Proportion of households with at least one ITN for every two people among households owning any ITN (P7)	Number of households owning at least one ITN for every two household members	Number of households owning at least one ITN
Intra-ownership gap, the proportion of households owning less than one ITN for every two household members, is calculated as 1-P7		
Use		
Proportion of population sleeping under an ITN the previous night (P4)	Number of household members who slept under an ITN the night before the survey	Population in the survey
Proportion of children under 5 years sleeping under an ITN the previous night (P5)	Number of children under 5 years who slept under an ITN the night before the survey	Number of children under five years in surveyed households
Proportion of pregnant women sleeping under an ITN the previous night (P6)	Number of pregnant women who slept under an ITN the night before the survey	Number of pregnant women in surveyed households
Proportion of population sleeping under an ITN the previous night among those with access (P8)	Number of household members who slept under an ITN the night before the survey	Total number of people with access to an ITN, calculated as the sum of all access (P3) values
Behavioural gap, the proportion of household members who did not sleep under an ITN despite having access to one, is calculated as 1-P8		

### Statistical methods

Data was analysed using Stata version 14 software and the maps were made using the R software. Point estimates (in percentage) and 95% confidence intervals were computed for each indicator and data point. In addition the percentage point changes between the baseline (2003) and endline (2014) were computed to assess change in the indicator and statistical significance assess at 5% level. Change by region and socio-demographical factor of each indicator between 2003 and 2014 were explored using the difference between weighted proportions (with svy prop command for survey data analysis) in Stata version 14 followed by a Lincom command (Linear combination of estimators). The survey mean command followed by Lincom (to compute two-sample t-test for difference in means with sampling weights) was used for the continuous variable access. Using this approach, we were directly testing whether the observed difference was significantly superior to zero.

## Results

### ITN ownership at household level (referred to as percentage 1-P1)

**Fig. 1:**
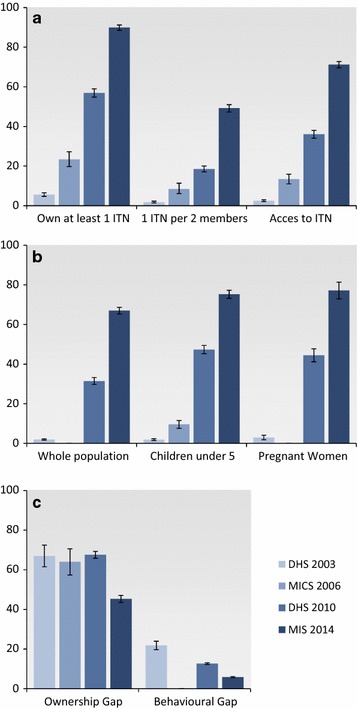
Trends of main malaria prevention indicators between 2003 and 2014, Burkina Faso. *Panel A* (ITN ownership) represents the proportion of households owning at least 1 ITN, owning 1 ITN for 2 household members and the proportion of individuals with access to an IT. *Panel B* (ITN use) represents the proportion of people who slept under an ITN the night before the survey for the whole population, among children under five years old and among pregnant women. *Panel C* (gap indicators) represents the proportion of household onwing at least 1 ITN, but less than 1 for every 2 household members (intraownership gap), and the proportion of people having access to an ITN but who did not use it the night before the survey (behavioural gap). All indicators are *plotted* for the 2003, 2006, 2010 and 2014 surveys, along with their 95% confidence interval

Respectively, 9097, 6034, 14,424, and 6448 households were visited in the DHS 2003, MICS 2006, DHS 2010, and MIS 2014. Ownership of ITN at household level in Burkina Faso was 5.6, 95% CI (4.7, 6.5%) in 2003, 23.3% (19.8, 27.3%) in 2006, 56.9% (54.8, 59.0%) in 2010, compared to 89.9% (88.5, 91.2%) in 2014 (Fig. 1). Overall ownership of ITNs at household level increased significantly from 2003 to 2014 (p < 0.001, Fig. 1). Ownership of ITNs in rural areas increased from 3.2% in 2003 to 90.8% in 2014 (p < 0.001). In urban areas, a percentage point increase of 72.5 of ITN ownership by households was observed from 2003 to 2014 (p < 0.001). In 2003, the richest households had the highest level of ITN ownership (15.8 vs 1.8% for poorest households). In 2014, ITN ownership increased and reached 84.4% in the poorest quintile compared to 87.4% in the richest quintile (Table 2).

**Table 2:** Proportion of households owning at least one insecticide-treated bed net

Background characteristic	DHS 2003		MICS 2006		DHS 2010		MIS 2014		Percentage point change (2003–2014)
	% (95% CI)	N	% (95% CI)	N	% (95% CI)	N	% (95% CI)	N	
Residence									
Urban	14.8 (12.5–17.2)	2182	45.0 (38.6–51.4)	921	60.0 (56.9–63.0)	4391	87.8 (85.2–90.4)	1305	72.5 (69.0–76.0)
Rural	3.2 (2.4–4.0)	6868	14.9 (12.8–17.1)	4602	55.9 (53.3–58.5)	9997	90.8 (89.2–92.4)	5104	87.6 (85.8–89.4)
Household wealth quintiles									
Poorest	1.8 (0.7–3.0)	1880	8.4 (6.4–10.5)	1276	48.8 (44.9–52.7)	2620	84.4 (81.0–87.7)	1511	82.6 (79.0–86.2)
Poorer	2.6 (1.6–3.7)	1619	13.3 (10.5–16.1)	1276	53.4 (50.2–56.7)	2744	91.8 (90.2–93.4)	1385	89.0 (87.2–90.9)
Average	2.8 (1.8–3.8)	2023	14.1 (10.7–17.4)	1116	56.8 (53.7–60.0)	2777	93.8 (92.2–95.4)	1288	91.1 (89.2–92.9)
Richer	3.8 (2.4–5.2)	1407	23.5 (18.9–28.1)	1013	59.5 (56.4–62.7)	2922	94.0 (91.5–96.4)	1236	90.1 (87.3–92.8)
Richest	15.8 (13.3–18.3)	2121	52.1 (45.7–58.4)	842	65.1 (62.3–67.9)	3325	87.4 (84.9–89.9)	989	70.8 (67.1–74.4)
Size of the household									
Small (1–5 members)	6.0 (4.7–7.2)	4421	24.5 (19.4–29.7)	2345	53.3 (51.1–55.6)	8169	88.3 (86.6–90.0)	3362	82.0 (79.8–84.1)
Medium (6–8 members)	5.5 (4.1–6.8)	2401	22.7 (18.9–26.5)	1665	60.3 (57.8–62.8)	3911	91.4 (89.6–93.2)	1760	85.9 (83.6–88.2)
Lager (9+ members)	4.8 (3.7–5.9)	2228	21.9 (17.4–26.3)	1513	63.7 (60.8–66.7)	2308	92.6 (90.8–94.5)	1287	87.8 (85.7–89.9)

All estimates take into account sample weights

*CI* Confidence intervals, *DHS* Demographic and Health Survey, *MICS* Multiple Clusters Indicator Survey, *MIS* Malaria indicator Survey, *N* number of households

**Fig. 2:**
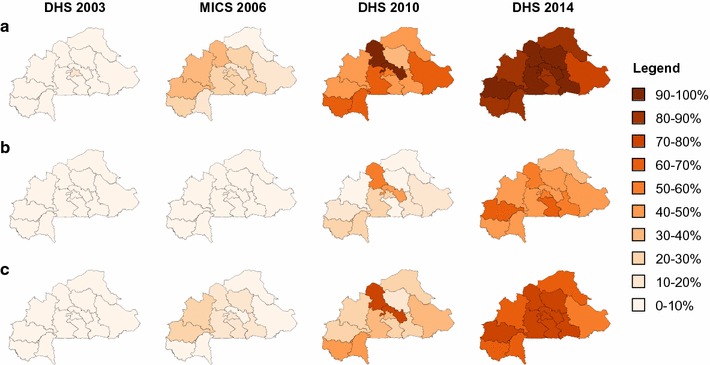
Trends of main ownership indicators between 2003 and 2014, Burkina Faso. The proportion of households owning at least 1 ITN (**a**), owning at least 1 ITN for 2 household members (**b**) and the proportion of the population with access to an ITN (**c**) is indicated for each region, for 2003, 2006, 2010, and 2014, respectively

Insecticide-treated bed nets coverage also increased significantly in different regions from 2003 to 2014. The percentage point increases were consistently high across regions (from 76.9 to 94.5). Compared to 2003 and 2010, ITN ownership was rather stable across the country and displayed limited geographic heterogeneity in 2014 (Fig. 2).

### ITN ownership at household level (P2: households with at least one ITN for every two people)

The proportion of households with enough ITNs for every household member, i.e., at least one ITN for every two people, was 1.8% (1.4, 2.3%) in 2003, 8.4% (6.1, 11.4%) in 2006, 18.5% (17.1, 20.0%) in 2010, compared to 49.2% (47.3, 51.0%) in 2014, indicating a substantial increase (p < 0.001). Household access to ITNs improved significantly from 2003 to 2014 in urban and rural areas, in all quintiles of wealth and in the different regions in Burkina Faso. The largest increases were observed in urban areas amongst the richest two quintiles, in smallest households and in the Hauts-Bassins and Central-South regions (Table 3). In these two regions, 62.5 and 60.4% households, respectively, owned at least one ITN for every two members in 2014 (Fig. 2).

**Table 3:** Proportion of households owning at least one insecticide-treated bed net for every two members

Background characteristic	DHS 2003		MICS 2006		DHS 2010		MIS 2014		Percentage point change (2003–2014)
	% (95% CI)	N	% (95% CI)	N	% (95% CI)	N	% (95% CI)	N	
Residence									
Urban	5.8 (4.2–7.4)	2182	20.9 (16.2–25.7)	921	24.8 (22.5–27.1)	4391	55.6 (52.4–58.8)	1305	49.0 (45.4–52.6)
Rural	0.8 (0.5–1.1)	6868	3.6 (2.7–4.4)	4602	16.4 (14.6–18.1)	9997	46.5 (44.4–48.5)	5104	45.4 (43.3–47.5)
Household wealth quintile									
Poorest	0.6 (0.1–1.1)	1880	1.5 (0.8–2.2)	1276	12.4 (10.3–14.5)	2620	41.1 (37.0–45.1)	1511	40.3 (36.3–44.4)
Poorer	0.2 (0.0–0.5)	1619	2.4 (1.3–3.4)	1276	15.4 (13.2–17.7)	2744	47.4 (44.2–50.7)	1385	47.0 (43.8–50.2)
Average	0.5 (0.2–0.9)	2023	3.7 (2.3–5.0)	1116	17.0 (14.7–19.2)	2777	45.6 (42.3–48.8)	1288	44.8 (41.5–48.1)
Richer	0.8 (0.3–1.3)	1407	5.3 (3.5–7.0)	1013	17.7 (15.4–20.0)	2922	52.2 (48.7–55.6)	1236	51.0 (47.6–54.5)
Richest	6.5 (4.8–8.1)	2121	25.8 (21.0–30.6)	842	28.6 (26.2–31.0)	3325	58.3 (55.0–61.6)	989	50.9 (47.2–54.5)
Size of the household									
Small (1–5 members)	3.3 (2.5–4.0)	4421	14.4 (10.2–18.6)	2345	26.7 (24.7–28.7)	8169	65.7 (63.6–67.9)	3362	61.5 (59.3–63.8)
Medium (6–8 members)	0.7 (0.1–1.2)	2401	5.3 (3.6–7.0)	1665	9.8 (8.5–11.2)	3911	37.2 (34.1–40.2)	1760	36.5 (33.3–39.7)
Large (9+ members)	0.1 (0.0–0.3)	2228	1.2 (0.6–1.7)	1513	4.2 (3.1–5.3)	2308	17.9 (15.3–20.4)	1287	17.8 (15.2–20.3)

All estimates take into account sample weights

*CI* Confidence intervals, *DHS* Demographic and Health Survey, *MICS* Multiple Clusters Indicator Survey, *MIS* Malaria indicator Survey; *N* number of households

### Access to ITN at population level (P3)

Access to ITNs increased significantly from 2.5% (2.1, 3.0%) in 2003, to 13.4% (11.0, 15.9%) in 2006, 36.1% (34.1, 38.0%) in 2010, and reached 71.2% (69.6, 72.8%) in 2014 (p < 0.001) (Table 4; Fig. 1).

**Table 4:** Proportion of population having access to an insecticide-treated bed net

Background characteristic	DHS 2003		MICS 2006		DHS 2010		MIS 2014		Percentage point change (2003–2014)
	% (95% CI)	N	% (95% CI)	N	% (95% CI)	N	% (95% CI)	N	
Residence									
Urban	8.1 (6.6–9.6)	12,313	28.8 (23.4–34.1)	5691	40.2 37.3–43.0)	21,758	71.1 (6 7.8–74.4)	6733	63.0 (59.4–66.7)
Rural	1.3 (0.9–1.7)	46,530	8.5 (7.1–9.9)	32,813	34.9 (32.6–37.2)	58,774	71.2 (69.4–73.0)	31,660	69.9 (68.0–71.8)
Household wealth quintiles									
Poorest	0.7 (0.3–1.2)	10,802	4.3 (3.1–5.5)	8734	29.5 (26.4–32.6)	15,243	63.0 (59.7–66.4)	8380	62.3 (58.9–65.7)
Poorer	0.9 (0.5–1.2)	11,113	7.8 (5.8–9.7)	8072	33.5 (30.8–36.3)	15,389	72.3 (70.2–74.3)	8495	71.4 (69.3–73.5)
Average	1.1 (0.4–1.8)	14,345	8.2 (6.2–10.3)	8685	35.8 (33.0–38.5)	16,306	74.0 (72.0–76.0)	8520	72.8 (70.7–75.0)
Richer	1.5 (0.8–2.2)	10,056	12.0 (9.2–14.8)	7702	37.5 (34.8–40.2)	16,989	74.8 (72.4–77.3)	8165	73.3 (70.7–75.9)
Richest	8.3 (6.8–9.7)	12,527	34.9 (29.5–40.2)	5311	44.1 (41.4–46.7)	16,605	71.8 (68.1–75.6)	4833	63.5 (59.5–67.6)
Size of the household									
Small (1–5 members)	4.2 (3.2–5.1)	14,278	19.1 (15.0–23.2)	8166	42.2 (40.0–44.4)	27,130	79.7 (77.9–81.4)	11,250	75.5 (73.5–77.5)
Medium (6–8 members)	2.7 (1.9–3.5)	16,500	14.1 (11.4–16.7)	11,415	36.2 (34.0–38.3)	26,634	73.1 (71.0–75.2)	12,025	70.4 (68.1–72.7)
Large (9+ members)	1.5 (1.0–1.9)	28,065	10.2 (7.9–12.5)	19,434	29.8 (27.6–32.1)	26,768	62.7 (60.4–64.9)	15,118	61.2 (58.9–63.5)

All estimates take into account sample weights

*CI* Confidence intervals, *DHS* Demographic and Health Survey, *MICS* Multiple Clusters Indicator Survey, *MIS* Malaria indicator Survey, *N* number of individuals

### Use of ITN at individual level (P4)

In 2003, 2.0% (1.6, 2.3%) slept under a net. In 2010, the proportion of people who slept under an ITN was 31.5% (29.8, 33.2%) compared to 67.0% (65.3, 68.7%) in 2014, suggesting a considerable increase of 65.0% points from 2003 to 2014 (p < 0.001, Fig. 1). In urban areas, 5.6% of individuals used ITNs in 2003, a proportion that increased to 61.8% by 2014 (p < 0.001). In rural areas, a significant increase was also observed, with 1.2% of people who used an ITN in 2003 and 68.8% in 2014 (p < 0.001). The substantial increase in the proportion of people who slept under ITNs was observed across all quintiles of wealth. Use of ITNs increased significantly from 0.8% in 2003 to 63.7% in 2014 (p < 0.001) in the poorest wealth quintile. In the second poorest wealth quintile, ITN use increased from 0.8% in 2003 to 69.1% in 2014 (p < 0.001), compared to an increase of 54.9% points in the richest quintile (6.1–61.0%) (Table 5).

**Table 5:** Proportion of population who slept under an insecticide-treated bed net the night before the survey

Background characteristic	DHS 2003		MICS 2006^a^		DHS 2010		MIS 2014		Percentage point change (2003–2014)
	% (95% CI)	N	% (95% CI)	N	% (95% CI)	N	% (95% CI)	N	
Residence									
Urban	5.6 (4.4–6.7)	12,313	–	–	31.2 (28.0–34.5)	21,758	61.8 (58.8–64.8)	6733	56.2 (53.0–59.4)
Rural	1.2 (0.8–1.5)	46,530	–	–	31.6 (29.5–33.6)	58,774	68.8 (67.0–70.6)	31,660	67.6 (65.8–69.5)
Household wealth quintiles									
Poorest	0.8 (0.3–1.2)	10,802	–	–	25.9 (23.4–28.5)	15,243	63.7 (60.5–66.8)	8380	62.9 (59.7–66.1)
Poorer	0.8 (0.4–1.1)	11,113	–	–	30.2 (27.7–32.7)	15,389	69.1 (66.9–71.3)	8495	68.3 (66.1–70.5)
Average	0.9 (0.4–1.5)	14,345	–	–	32.7 (30.2–35.1)	16,306	71.2 (68.8–73.5)	8520	70.2 (67.8–72.7)
Richer	1.3 (0.7–1.9)	10,056	–	–	34.5 (31.8–37.1)	16,989	70.1 (67.6–72.6)	8165	68.8 (66.2–71.4)
Richest	6.1 (4.8–7.3)	12,527	–	–	34.2 (31.1–37.3)	16,605	61.0 (57.8–64.2)	4833	54.9 (51.5–58.3)
Size of the household									
Small (1–5 members)	3.1 (2.5–3.8)	14,278	–	–	37.3 (35.2–39.3)	27,130	72.3 (70.3–74.4)	11,250	69.2 (67.0–71.3)
Medium (6–8 members)	2.1 (1.4–2.8)	16,500	–	–	31.0 (29.0–33.0)	26,634	69.5 (67.4–71.6)	12,025	67.4 (65.2–69.7)
Large (9+ members)	1.2 (0.8–1.7)	28,065	–	–	26.2 (24.1–28.4)	26,768	60.6 (58.1–63.0)	15,118	59.4 (56.8–61.9)

All estimates take into account sample weights

*CI* Confidence intervals, *DHS* Demographic and Health Survey, *MICS* Multiple Clusters Indicator Survey, *MIS* Malaria indicator Survey, *N* number of individuals

^a^Data not available

**Fig. 3:**
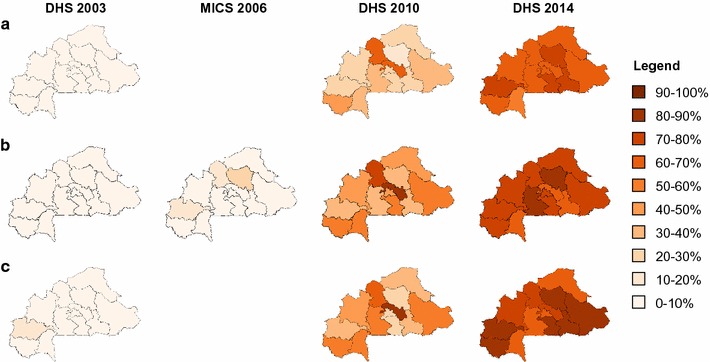
Trends of main usage indicators between 2003 and 2014, Burkina Faso. The proportion of the population (**a**), of children under 5 years (**b**) and pregnant women (**c**) who slept under an ITN the night before the survey is indicated for each region, for 2003, 2006, 2010 and 2014 respectively

In terms of regions, the largest increase occurred in the Central-East and Central-South regions. The absolute increase in ITN use in the Central-East region was 75.9% points (p < 0.001), increasing from 2.6% in 2003 to 78.6% in 2014. The corresponding estimates for the Central-South region were 1.4% in 2003 and 75.4% in 2014 (p < 0.001) (Fig. 3).

### Use of ITN among children under 5 years of age (P5)

In 2003, 1.9% (1.5, 2.4%) of children under 5 years of age were sleeping under an ITN, compared to 9.6% (7.6, 11.6%) in 2006, 47.4% (45.3, 49.5%) in 2010, and 75.2% (73.2, 77.3%) in 2014 (Table 5). Overall, the use of ITNs among children under five years has increased significantly from 2003 to 2014 (p < 0.001) (Fig. 1).

Analysis of ITN use by age band showed a significant increase from 2003 to 2014. In children younger than 12 months, use of ITNs increased from 1.9% in 2003 to 77.0% in 2014 (p < 0.001). Among children aged 12–23 months, the proportion that used ITNs increased from 2.2% in 2003 to 76.7% in 2014, suggesting an absolute increase of 74.5% points between the two periods (p < 0.001). Substantial increases in the use of ITNs also were observed in older children (ages 24, 36 and 48 months) from 2003 to 2014 (Table 6).

**Table 6:** Proportion of children under 5 years old who slept under an insecticide-treated bed net the night before the survey

Background characteristic	DHS 2003		MICS 2006		DHS 2010		MIS 2014		Percentage point change (2003–2014)
	% (95% CI)	N	% (95% CI)	N	% (95% CI)	N	% (95% CI)	N	
Age group (months)									
0–11	1.9 (1.2–2.6)	2153	11.3 (8.6–13.9)	1209	55.6 (52.4–58.8)	1517	77.0 (73.8–80.2)	1412	75.1 (71.8–78.4)
12–23	2.2 (1.5–2.9)	1892	8.1 (5.7–10.4)	1069	54.6 (51.1–58.1)	1427	76.7 (73.8–79.5)	1314	74.5 (71.6–77.4)
24–35	2.3 (1.4–3.2)	1819	10.2 (7.6–12.9)	1149	46.3 (42.7–49.9)	1426	74.7 (71.9–77.5)	1408	72.4 (69.5–75.3)
36–47	1.9 (1.1–2.7)	2091	10.0 (5.0–15.0)	1060	45.4 (42.1–48.7)	1447	75.5 (72.7–78.3)	1419	73.6 (70.7–76.6)
48–59	1.4 (0.7–2.1)	1867	7.7 (4.5–10.8)	796	45.2 (42.9–47.5)	8407	72.4 (69.1–75.7)	1369	70.9 (67.5–74.3)
Gender									
Male	1.8 (1.3–2.3)	5061	10.2 (7.5–12.9)	2677	47.9 (45.7–50.2)	7223	75.5 (73.3–77.8)	3506	73.7 (71.4–76.0)
Female	2.1 (1.5–2.6)	4761	9.0 (6.9–11.2)	2604	46.8 (44.4–49.1)	7001	75.0 (72.5–77.4)	3416	72.9 (70.3–75.4)
Residence									
Urban	6.2 (4.4–7.9)	1631	23.8 (17.8–29.9)	573	45.5 (41.5–49.6)	3167	69.9 (64.8–75.1)	1051	63.7 (58.3–69.2)
Rural	1.3 (0.9–1.8)	8191	6.2 (4.7–7.8)	4710	47.8 (45.4–50.1)	11,057	76.8 (74.7–78.8)	5871	75.5 (73.4–77.6)
Household wealth quintiles									
Poorest	1.2 (0.3–2.2)	1810	4.4 (2.5–6.4)	1255	41.2 (37.4–45.0)	2756	72.3 (68.4–76.3)	1528	71.1 (67.0–75.3)
Poorer	0.7 (0.2–1.3)	2011	6.0 (3.7–8.3)	1100	46.4 (43.0–49.8)	2923	76.1 (73.1–79.0)	1570	75.3 (72.3–78.3)
Average	0.8 (0.3–1.2)	2582	6.0 (3.8–8.2)	1306	49.2 (46.0–52.3)	3109	78.5 (75.4–81.6)	1604	77.7 (74.6–80.9)
Richer	1.6 (0.6–2.6)	1748	9.4 (6.2–12.7)	1091	51.5 (48.1–54.9)	3086	78.3 (74.4–82.2)	1508	76.7 (72.7–80.7)
Richest	7.0 (4.9–9.1)	1671	26.2 (21.5–30.9)	531	48.8 (45.1–52.6)	2350	69.7 (64.3–75.1)	712	62.7 (56.9–68.5)
Size of the household									
Small (1–5 members)	2.9 (1.9–3.9)	2365	15.4 (9.8–21.0)	1039	53.5 (51.1–55.9)	4853	77.9 (74.5–81.4)	2135	75.1 (71.5–78.7)
Medium (6–8 members)	2.2 (1.4–3.1)	2633	10.0 (6.6–13.5)	1590	46.4 (43.6–49.1)	4634	78.2 (75.4–81.1)	2048	76.0 (73.0–79.0)
Large (9+ members)	1.3 (0.7–1.9)	4824	6.5 (4.8–8.3)	2654	42.0 (39.0–45.1)	4737	70.7 (67.9–73.5)	2739	69.4 (66.6–72.3)

All estimates take into account sample weights

*CI* Confidence intervals, *DHS* Demographic and Health Survey, *MICS* Multiple Clusters Indicator Survey, *MIS* Malaria indicator Survey, *N* number of children

In urban settings, 6.2% of children under five years slept under an ITN in 2003, compared to 69.9% in 2014, indicating an absolute increase of 63.7% points (p < 0.001). In 2003, 1.3% of children under 5 years living in rural areas slept under an ITN. This proportion increased significantly in 2014, reaching 76.8% in children under 5 years living in rural areas (p < 0.001).

In wealth quintiles, the smallest increases were observed in children under 5 years from the richest wealth quintile, with an increase from 7.0% in 2003 to 69.7% in 2014 (Table 6).

Marked increases in ITN use were also achieved in all regions over the specified period; however, the Centre-East and Centre-Nord regions displayed the greatest increase in ITN use compared to the other regions with an increase from 2.5 to 86.5% and from 0.5 to 82.1% (Table 6, Fig. 3).

### Use of ITN among pregnant women (P6)

The use of ITNs by pregnant women in Burkina Faso was 3.0% (1.9, 4.2%) in 2003, 44.5% (41.2, 49.%) in 2010, and 77.1% (72.9%, 81.3%) in 2014, indicating a significant increase from 2003 to 2014 (p < 0.001) (Fig. 1). In urban areas, 7.5 and 69.6% of pregnant women used ITNs in 2003 and 2014, respectively, an increase of 62.1% points (p < 0.001, Table 7). The corresponding estimates in rural areas were 2.1% in 2003 and 78.8% in 2014, a significant improvement in ITN use among pregnant women between these periods (p < 0.001). A trend similar to that of ITN use in children under five years was found when analyses were performed by wealth quintile.

**Table 7:** Proportion of pregnant women who slept under an insecticide-treated bed net the night before the survey

Background characteristic	DHS 2003		MICS 2006^a^		DHS 2010		MIS 2014		Percentage point change (2003–2014)
	% (95% CI)	N	% (95% CI)	N	% (95% CI)	N	% (95% CI)	N	
Residence									
Urban	7.5 (4.0–10.9)	219	–	–	38.3 (30.7–45.9)	378	69.6 (57.5–81.8)	6733	62.1 (49.6–74.7)
Rural	2.3 (1.1–3.6)	1056	–	–	45.8 (42.2–49.4)	1310	78.8 (74.5–83.1)	31,660	76.5 (72.0–80.9)
Household wealth quintiles									
Poorest	1.1 (−0.4–2.7)	221	–	–	44.0 (37.5–50.5)	297	69.0 (60.8–77.2)	161	67.9 (59.6–76.2)
Poorer	0.7 (−0.4–1.7)	269	–	–	42.5 (36.4–48.6)	369	78.5 (71.4–85.6)	8495	77.8 (70.7–84.9)
Average	3.2 (0.4–5.9)	343	–	–	43.2 (37.1–49.2)	365	79.8 (72.9–86.8)	8520	76.7 (69.3–84.1)
Richer	1.5 (−0.4–3.3)	216	–	–	49.1 (42.4–55.7)	358	87.8 (82.1–93.6)	8165	86.4 (80.4–92.4)
Richest	9.4 (4.9–14.0)	226	–	–	43.9 (36.2–51.6)	299	65.5 (50.8–80.3)	4833	57.4 (42.2–72.5)
Size of the household									
Small (1–5 members)	4.3 (2.3–6.3)	442	–	–	47.0 (42.6–51.4)	813	75.8 (69.7–81.9)	11,250	71.5 (65.1–77.9)
Medium (6–8 members)	2.0 (0.0–4.0)	350	–	–	42.1 (36.9–47.2)	463	78.3 (71.5–85.2)	12,025	76.3 (69.2–83.4)
Large (9+ members)	2.5 (0.3–4.7)	483	–	–	42.2 (36.3–48.1)	412	78.4 (71.3–85.6)	15,118	75.9 (68.5–83.4)

All estimates take into account sample weights

*CI* Confidence intervals, *DHS* Demographic and Health Survey, *MICS* Multiple Clusters Indicator Survey, *MIS* Malaria indicator Survey, *N* number of pregnant women

^a^Data not available

### ITN ownership and use gaps

In 2003, 94.4% (93.5, 95.2%) of the study households did not possess an ITN (Fig. 1). Among those who owned at least one ITN, 67.0% (61.5, 72.4%) did not have sufficient bed nets to protect all members (intra-household net ownership gap). However, 19.4% (n = 94) of these households had excess ITNs (i.e., more than one ITN for every two people). A significant proportion (21.9%, n = 316) of the population with sufficient access to ITNs did not actually use them the night before the survey.

In 2010, 43.0% (41.0, 45.2%) of the study households did not have an ITN. The intra-household net ownership gap was 67.6% (65.8, 69.3%), indicating that about two-thirds of the households with at least one ITN did not have sufficient ITNs to protect all members. This gap is presented in Table 8 by background characteristics and shows that the gap was very high in large household size (93.4%) and rural areas (70.4%). However, 18.3% (n = 1475) of these households had excess ITNs (i.e., more than one ITN for every two people). A small proportion (12.7%, n = 3700) of the population with sufficient access to ITNs did not actually use them.

**Table 8:** Proportion of households owning at least one insecticide-treated bed net but with fewer than one net for every two household members (intra-ownership gap)

Background characteristic	DHS 2003		MICS 2006		DHS 2010		MIS 2014		Percentage point change (2003–2014)
	% (95% CI)	N	% (95% CI)	N	% (95% CI)	N	% (95% CI)	N	
Residence									
Urban	60.8 (53.8–67.8)	2182	53.5 (45.1–62.0)	921	58.7 (56.0–61.3)	4391	36.7 (33.5–39.8)	1305	23.9 (15.8–31.9)
Rural	74.4 (66.8–81.9)	6868	76.2 (72.8–79.6)	4602	70.7 (68.5–72.9)	9997	48.9 (47.0–50.7)	5104	25.9 (18.2–33.7)
Household wealth quintiles									
Poorest	67.0 (51.0–82.9)	1880	82.5 (75.0–90.1)	1276	74.6 (71.4–77.8)	2620	51.3 (47.4–55.2)	1511	15.4 (−0.8–31.6)
Poorer	90.5 (79.6–101.4)	1619	82.2 (76.0–88.5)	1276	71.1 (67.9–74.3)	2744	48.3 (45.1–51.5)	1385	42.1 (30.3–53.8)
Average	80.4 (67.7–93.2)	2023	74.0 (67.1–80.9)	1116	70.2 (67.2–73.2)	2777	51.4 (48.1–54.7)	1288	28.7 (15.6–41.9)
Richer	79.4 (66.6–92.2)	1407	77.6 (71.9–83.3)	1013	70.2 (67.2–73.3)	2922	44.5 (41.1–47.9)	1236	35.2 (22.7–47.8)
Richest	59.2 (52.8–65.6)	2121	50.4 (42.9–57.9)	842	56.1 (53.4–58.8)	3325	33.3 (30.2–36.4)	989	26.0 (18.8–33.2)
Size of the household									
Small (1–5 members)	45.2 (38.2–52.2)	4421	41.2 (34.9–47.5)	2345	49.9 (47.8–52.1)	8169	25.5 (23.7–27.4)	3362	20.4 (13.3–27.5)
Medium (6–8 members)	87.9 (78.3–97.5)	2401	76.6 (69.7–83.6)	1665	83.7 (81.7–85.7)	3911	59.3 (56.2–62.5)	1760	28.6 (18.0–39.2)
Large (9+ members)	97.5 (94.5–100.5)	2228	94.7 (91.8–97.6)	1513	93.4 (91.7–95.1	2308	80.7 (78.0–83.4)	1287	16.8 (12.9–20.8)

All estimates take into account sample weights

*CI* Confidence intervals, *DHS* Demographic and Health Survey, *MICS* Multiple Clusters Indicator Survey, *MIS* Malaria indicator Survey, *N* number of households

In 2014, only 10.6% (9.2, 12.0%) of the study households did not have an ITN. The intra-household net ownership gap was 45.3% (43.6, 47.1%), indicating that about half of the households with at least one ITN did not have sufficient ITNs to protect all members. This gap was 80.7% in large household size and well above the national average (Table 8). However, 34.5% (n = 1926) of these households had excess ITNs (i.e., more than one ITN for every two people). A small proportion (5.9%, n = 1562) of the population with sufficient access to ITNs did not actually use them. In contrast, this proportion was 13.1% in urban areas and only 3.4% in rural areas (Table 9).

**Table 9:** Proportion of individuals with access to an insecticide-treated bed net who did not use them the night before the survey (behavioural gap)

Background characteristic	DHS 2003		MICS 2006^a^		DHS 2010		MIS 2014	
	% (95% CI)	N	% (95% CI)	N	% (95% CI)	N	% (95% CI)	N
Residence								
Urban	31.0 (27.9–34.1)	12,313	–	–	22.2 (21.2–23.2)	21,758	13.1 (12.3–13.9)	6733
Rural	9.6 (7.3–11.9)	46,530	–	–	9.6 (9.2–10.0)	58,774	3.4 (3.1–3.7)	31,660
Household wealth quintiles								
Poorest	0.0^b^ (0.0–1.2)	10,802	–	–	12.0 (11.1–12.9)	15,243	0.0^b^ (0.0–0.0)	8380
Poorer	8.9 (3.2–14.6)	11,113	–	–	9.9 (9.1–10.7)	15,389	4.4 (3.9–4.9)	8495
Average	17.8 (11.9–23.7)	14,345	–	–	8.6 (7.9–9.3)	16,306	3.8 (3.3–4.3)	8520
Richer	16.9 (10.9–22.9)	10,056	–	–	8.1 (7.4–8.8)	16,989	6.4 (5.8–7.0)	8165
Richest	26.6 (23.8–29.4)	12,527	–	–	22.5 (21.5–23.5)	16,605	15.0 (14.0–16.0)	4833
Size of the household								
Small (1–5 members)	24.6 (21.2–28.0)	14,278	–	–	11.7 (11.1–12.3)	27,130	9.2 (8.6–9.8)	11,250
Medium (6–8 members)	23.0 (19.1–26.9)	16,500	–	–	14.3 (13.6–15.0)	26,634	4.9 (4.4–5.4)	12,025
Large (9+ members)	16.3 (12.7–19.9)	28,065	–	–	12.1 (11.4–12.8)	26,768	3.3 (2.9–3.7)	15,118

All estimates take into account sample weights

*CI* Confidence intervals, *DHS* Demographic and Health Survey, *MICS* Multiple Clusters Indicator Survey, *MIS* Malaria indicator Survey, *N* number of individuals

^a^Data not available

^b^Negative gap values were set to zero

## Discussion

The Government of Burkina Faso set a national goal to increase ITN ownership, access and use. These data provide evidence of the remarkable increase in the coverage of ITN ownership, particularly in 2014 after the second free distribution campaign. Indeed, ownership, access and use indicators calculated following MERG’s recommendations [21] dramatically increased between 2003 and 2014 and was particularly successful at reaching the poorest populations. The increasing trend in ITN ownership described here, is consistent with data from 19 SSA countries during a similar time period [26]. The data show that the two free distribution campaigns substantially increased ITN ownership and reduced inequity among populations in Burkina Faso. These findings are consistent with other free mass distribution campaigns that have been carried out in SSA [9, 27], demonstrating that this strategy can be used to rapidely scale-up ITN coverage in areas with low coverage and reduce social inequity. However, despite the significant progress, less than 50% of households own enough ITNs to protect every household members (Fig. 1). These campaigns should not represent the only mechanism by which ITNs are distributed to poorest communities and vulnerable populations [9]. In Burkina Faso, ITNs were provided for free to pregnant women and children under five years of age through routine channels, such as antenatal care and immunization campaigns. ITNs were also available for purchase in retail shops and stores. This could explain both the slight increase in bed net ownership (Fig. 1) and use (Fig. 3). However, the relative contribution of these distribution channels remains, to date, very limited. More than 90% of the ITNs were obtained during the free distribution campaign [25]. To reach and maintain high ITN coverage in Burkina Faso, there is a need to improve the contribution of the routine distribution through ANC and vaccination programs and develop alternative strategies, such as the continuous distribution of ITN in schools and by community health workers for replacement [28]).

**Fig. 4:**
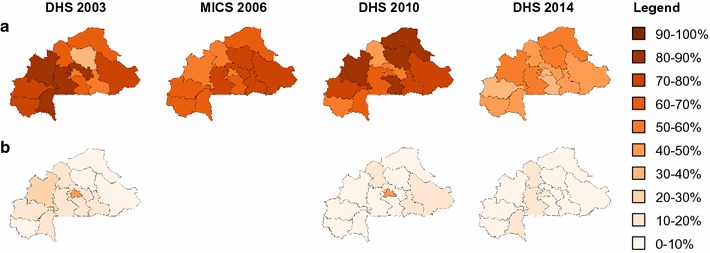
Trends of ownership and behavioural gaps between 2003 and 2014, Burkina Faso. The proportion of households owning at least 1 ITN but less than 1 for every 2 members (**a**) and the proportion of people with access to ITN but who did not use it the night before the survey (**b**) is indicated for each region, for 2003, 2006, 2010 and 2014 respectively

Ownership and behavioural gap analyses provide complementary information regarding ITN ownership and use. The results reveal geographical and sociogeographic discrepancies of ITN ownership and use. Gap decreased (>10% point change) in all regions, with highest decreases in the Hauts-Bassins and, in the Sud-Ouest half of the country (zones where malaria transmission is permanent with a peak during the rainy season). Sahel, Nord and Centre-Est (where malaria transmission is seasonal) display lower gap reduction, but the ownership gap was already low in 2003. Change in malaria transmission may explain this difference. In 2014, geographical discrepancies in the ownership gap were minimum. Remarkably, the ownership gap increased in Centre-Nord. This increase can be attributed to the 2003 gap value, which is clearly an outlier: 35.8% gap, while the gap in all other regions fell between 59 and 90 (Fig. 4).

By contrast with ownership gap, behavioural gap remained stable across the country. However, significant decrease was observed in Boucles de Mouhoun, Centre, Centre-Sud, and Centre-Est. All four regions displayed higher-than-average behavioural gap in 2003. In this respect, Centre consistently displayed higher behavioural gap values for all years studied, most likely because the population in this region is concentrated in urban habitat (Ouagadougou). This result could be explained by higher population dynamics in the capital region. Interestingly also, the change in behaviour is very recent in this region (in 2010, behavioural gap was 40%). In 2014, the behavioural gap was uniformly low across the country (0–15%). This reduction is probably a result of the health promotion programmes initiated by the Government of Burkina Faso to improve awareness concerning malaria prevention methods [25].

Household size is the main factor associated with ownership gap in this study. Large households with at least one bed net lacked additional bed nets to protect all family members (vs 25.5% for small households). Conversely, households in urban settings and from the richest quintile of the population more frequently owned enough bed nets than households located in rural settings or with a lower wealth index. This result is consistent with the findings of other studies showing that ITN coverage is lower in urban areas because mass distribution campaigns usually focus on rural communities [29, 30]. Therefore, future strategies for ITN distribution should pay partiuclar attention to urban areas.

Overall, the behavioural gap was very low in 2014. However, households located in urban settings and from the richest quintile of wealth index have higher gaps, because they might have other alternative prevention methods, such as better housing. Also, behavioural gap was significantly lower for large households which could result from large households having more family members (especially children) sleeping under the same bed net. The results showed that only a few large households possessed enough bed nets to protect all family members.

This study has a few limitations, however, they do not affect the validity of the results. This study was based on exiting data, and was limited by available data. Survey data were collected during different seasons of the year. MIS data were collected during the high transmission period while DHS data were collected during the end of the transmission period. This difference could potentially affect the trends analysis and may have under- or overestimated the effect size, as ITN use can be seasonal depending on the perceived nuisance of mosquitoes [31]. Furthermore, the measures presented in this paper were self-reported and therefore susceptible to social desirability biases.

## Conclusion

Following the two free mass distribution campaigns in 2010 and 2013, Burkina Faso has made considerable progress in coverage of ITN ownership, access and use between 2003 and 2014. However, bed net coverage remains below national targets of 100% for ownership and 80% for use. To reduce significantly the malaria burden in Burkina Faso, the NMCP needs to increase further and sustained ITN ownership and use in the general population. The free mass distribution campaigns contributed effectively to increase INT ownership and use in Burkina Faso. The NMCP should continue implementing these campaigns to reach the universal coverage target. In addition, these campaigns should be complemented by other bed net distribution mechanisms (through antenatal care, immunization) to identify and replace nets that are worn, damaged or lost between free mass distribution campaigns. Furthermore, NMCP should have an effective behaviour change communication component in all distribution mechanisms to ensure that the population use bed nets consistently.

## Abbreviations

ANC: antenatal care
RBM: Roll Back Malaria
BBC: behaviour change communication
DHS: Demographic and Health Surveys
EA: enumeration areas
LLINs: long-lasting insecticidal nets
MERG: Monitoring and Evaluation Reference Group
MICS: Multiple Indicator Cluster Surveys
MIS: Malaria Indicator Survey
ITNs: insecticide-treated bed nets
NMCP: national malaria control programme
SSA: sub-Saharan Africa
WHO: World Health Organization
